# Context-dependent mTOR pharmacology in atherosclerotic plaque stability and exposure-guided modulation

**DOI:** 10.3389/fphar.2026.1994231

**Published:** 2026-10-26

**Authors:** Ruoyu Han, Mao Peng, Zihang Song, Yifan Tang, Ziyi Yao, Yan She, Shuhan Cheng, Xiaomin Li, Pingping He

**Affiliations:** 1 Key Laboratory of Model Animals and Stem Cell Biology in Hunan Province, Health Science Center, Hunan Normal University, Changsha, China; 2 Health Management Center, Hengyang Central Hospital Affiliated to Hunan Normal University, Hengyang, China; 3 Aging Health Research Center, School of Nursing, Health Science Center, Hunan Normal University, Changsha, China

**Keywords:** atherosclerosis, lesion-directed drug delivery, mTOR signalling, pharmacokinetics–pharmacodynamics, plaque stability, rapalogs, target engagement

## Abstract

Despite advances in lipid lowering, anti-inflammatory therapy and revascularization, residual cardiovascular risk may partly reflect persistent lesion-level inflammation and defective plaque repair that are not fully corrected by systemic risk-factor control. The mechanistic target of rapamycin (mTOR) integrates inflammatory, metabolic and reparative programmes, but its effects vary with cell state, plaque niche, complex identity, and signalling amplitude and duration. Evidence is strongest for context-dependent macrophage mTOR signalling, yet macrophage/myeloid mTORC2 disruption has produced opposing lesion phenotypes across genetic models; endothelial and smooth-muscle evidence remains less direct. Pharmacological, genetic and lesion-directed delivery studies demonstrate that mTOR perturbation can modify experimental atherosclerosis. However, genetic perturbation cannot be equated with rapalog pharmacology, and existing studies do not yet define cell-resolved exposure–engagement–response relationships and relate them to plaque composition. This evidential asymmetry limits interpretation of whether broad or prolonged mTOR inhibition can selectively suppress disease-amplifying outputs while preserving homeostatic programmes. In this Review, focused primarily on lipid-rich, rupture-prone plaques and using rapalogs as the principal pharmacological model, we apply an exposure-guided pharmacological framework to distinguish administered dose from lesion exposure, cellular availability, target engagement and pharmacodynamic recovery. Compartment-resolved PK–PD studies, cell-specific pathway interrogation and large-animal validation will be needed to quantify these relationships and determine whether maladaptive mTOR outputs can be suppressed without compromising vascular repair.

## 1 Introduction

Contemporary lipid-lowering, anti-inflammatory and revascularization strategies have substantially reshaped the management of atherosclerotic cardiovascular disease (ASCVD) (Sarraju and Nissen, 2024). In the HUYGENS coronary imaging trial, the addition of evolocumab to intensive statin therapy following non-ST-segment elevation myocardial infarction produced favourable changes in coronary plaque phenotype and burden (Nicholls et al., 2022). By contrast, the CANTOS and LoDoCo2 cardiovascular outcome trials demonstrated that IL-1β inhibition with canakinumab and low-dose colchicine, respectively, reduced cardiovascular event rates in selected secondary-prevention populations (Ridker et al., 2017; Nidorf et al., 2020). Nevertheless, residual cardiovascular risk persists despite effective risk-factor modification, reflecting the continued activity of inflammatory and reparative processes within established atherosclerotic lesions (Gluba-Brzózka et al., 2021; Soehnlein and Libby, 2021; Dimitroglou et al., 2023; Arnold and Koenig, 2025; Raone et al., 2026).

This residual risk has increased interest in the structural and biological features of the plaque beyond luminal stenosis alone. Intracoronary imaging studies have shown that high-risk lesions can be identified by features such as large plaque burden, lipid-rich or necrotic cores, and thin-cap fibroatheroma, even in the absence of severe luminal narrowing (Stone et al., 2011; Park et al., 2024). In advanced lipid-rich plaques, necrotic-core expansion and fibrous-cap thinning coexist with defective efferocytosis and endothelial dysfunction, processes that contribute to persistent inflammation and structural weakening (Bentzon et al., 2014; Basatemur et al., 2019; Xu et al., 2021; Adkar and Leeper, 2024; Fredman and Serhan, 2024; Jansen et al., 2024). These lesion-level abnormalities highlight the need to understand the signalling networks that integrate inflammatory, metabolic and reparative responses within the plaque.

The mechanistic target of rapamycin (mTOR) is a context-sensitive signalling node positioned at the intersection of metabolic sensing, autophagy, inflammatory signalling and vascular repair (Saxton and Sabatini, 2017; Panwar et al., 2023). mTOR assembles into two principal complexes, mTORC1 and mTORC2, which regulate largely overlapping but non-identical sets of downstream processes. This integrative position makes mTOR pharmacologically attractive, yet its regulation is biologically complex—perturbation of the same pathway can yield divergent outcomes depending on the duration of exposure (Panwar et al., 2023; Zhang et al., 2025). Its biological output cannot be inferred from pathway activity alone, because cell state, plaque niche, complex and substrate identity, and the magnitude and duration of pathway perturbation can each alter the consequences of pathway engagement (Sarbassov et al., 2006; Zhang et al., 2023). The same pathway may therefore support adaptive homeostasis in one setting while contributing to disease-amplifying processes in another. For a pleiotropic signalling node such as mTOR, the central biological question is not simply whether mTOR should be inhibited, but how a defined perturbation is translated into distinct cellular and lesion-level responses under specific biological contexts.

The pharmacological discussion in this Review uses rapamycin and rapalogs as the principal model for mTOR-directed intervention. These agents bind FK506-binding protein 12 (FKBP12) and inhibit mTOR allosterically, with acute effects weighted toward rapamycin-sensitive mTORC1 outputs, whereas prolonged exposure can impair mTORC2 assembly and protein kinase B (AKT) signalling in susceptible cellular contexts (Sarbassov et al., 2006; Zhang et al., 2025). This duration-dependent pharmacology differs from that of ATP-competitive mTOR kinase inhibitors, which engage the catalytic site and can acutely suppress outputs from both mTORC1 and mTORC2 regardless of exposure time (Zhang et al., 2025). Thus, the same nominal target does not imply equivalent complex selectivity, substrate coverage or temporal pharmacology. Accordingly, conclusions regarding duration dependence, complex selectivity and pathway recovery should not be generalized automatically across all mTOR-directed compounds. Throughout this Review, “mTOR perturbation” refers to genetic or pharmacological manipulation of mTOR biology, whereas “rapamycin/rapalogs” refers specifically to pharmacological inferences based on FKBP12-mediated allosteric inhibition.

Against this background, this Review asks how the context-dependent nature of plaque mTOR biology can be reconciled with pharmacological exposure (Zhang et al., 2023). To address this question, we distinguish three issues: which mTOR programmes are context dependent within plaques, what existing genetic and pharmacological studies actually demonstrate, and which measurements are required to define exposure–engagement–response relationships.

## 2 Context-dependent mTOR biology in atherosclerotic plaques

### 2.1 Plaque niches and cell-state dependence

The atherosclerotic plaque is best understood as a dynamic tissue ecosystem rather than as three isolated cellular compartments. Its luminal interface, inflammatory core and fibrous cap are coupled by lipid flux, cytokine exchange, cell migration and extracellular-matrix remodelling (Mahdinia et al., 2023; Gong et al., 2025). Endothelial dysfunction can alter leucocyte recruitment, macrophages contribute to the inflammatory milieu and its resolution, and smooth muscle cells (SMCs) adopt diverse phenotypically modulated states (Moore et al., 2018; Pan et al., 2020; Libby, 2021). Macrophage–SMC crosstalk further links inflammatory and structural features of the plaque (Yurdagul, 2022). We synthesize these observations into a niche-based interpretation of mTOR biology: spatial position and disease stage may shape the cellular context in which the pathway is engaged, but these observations do not directly establish niche-specific mTOR causality. Spatial niches tend to enrich for particular cell states, but this enrichment does not uniquely determine them, and multiple cellular states can coexist within the same lesion.

At the luminal interface, mTOR activity is embedded in the endothelial response to haemodynamic and inflammatory injury. Non-atherosclerotic vascular studies suggest that endothelial mTOR signalling can influence barrier integrity, nitric-oxide bioavailability and adaptive responses to vascular injury (Oberkersch et al., 2022; Wang et al., 2022; Millar et al., 2024). However, direct evidence linking endothelial-specific mTOR manipulation to spontaneous plaque outcomes remains sparse. Thus, the current data support a homeostatic and adaptive role for endothelial mTOR signalling but do not establish that either mTORC1 or mTORC2 is uniformly protective within atherosclerotic plaques. Endothelial pathway activity should instead be interpreted according to the specific injury context and the functional output being measured.

Within macrophage-rich regions, lipid accumulation and defective clearance of cellular debris are disease-relevant challenges, while nutrient excess can activate macrophage mTORC1 and impair mitophagy in experimental atherosclerosis (Moore et al., 2018; Zhang et al., 2020). We use lipid overload, nutrient excess, hypoxia, oxidative stress and impaired debris clearance as a contextual synthesis of potential plaque-associated challenges, rather than as an experimentally established set of direct mTOR inputs in every macrophage state. mTORC1 integrates nutrient and growth-related signals to regulate biosynthesis, autophagy and immune-cell differentiation (Powell et al., 2012; Byles et al., 2013). Disease-relevant studies have further linked macrophage mTORC1 activity to nutrient sensing and mitophagic control, with mTORC1 activation generally promoting a pro-inflammatory, lipid-accumulating phenotype that accelerates atherosclerotic progression (Zhang et al., 2020). Conversely, disruption of mTORC1 in macrophages reduces chemokine expression and attenuates lesion formation in experimental models (Ai et al., 2014). By contrast, mTORC2 loss in the myeloid lineage has yielded divergent outcomes: in lethally irradiated LDLR-deficient recipients reconstituted with bone marrow from mice carrying myeloid Rictor deficiency, mTORC2 disruption reduced lesion formation by impairing monocyte/macrophage proliferation and survival (Babaev et al., 2018); in contrast, in non-transplanted LysM-Cre/Rictor^flox/flox^/ApoE^−/−^ mice, mTORC2 loss drove larger and more complex lesions through FoxO1 derepression and IL-1β-dependent inflammatory signalling (Zhang et al., 2023). These studies differed not only in lesion stage but also in host genotype, irradiation and bone-marrow transplantation, and the experimental context in which Rictor was deleted. The opposing outcomes therefore cannot be attributed to disease stage alone. Although stage-dependent effects remain biologically plausible, this interpretation represents a hypothesis that requires direct testing in stage-matched experimental models. Single-cell studies identify heterogeneous plaque macrophage populations, including inflammatory and lipid-metabolism-associated states, rather than a uniform macrophage phenotype (Cochain et al., 2018; Fernandez et al., 2019). These atlases support state heterogeneity but do not, by themselves, establish a universal temporal transition from a proliferative recruited state to a lipid-laden, stress-adapted state, or identify the causal role of mTOR in such a transition. We therefore propose that macrophage mTOR output should be evaluated in relation to local stress and cell state; this is an interpretative synthesis, not a lineage-resolved trajectory demonstrated by these studies.

The fibrous cap further illustrates why lineage alone is an inadequate guide to mTOR function. SMCs provide the structural integrity of the cap, yet they can lose canonical contractile markers, adopt alternative phenotypes and contribute to foam-cell populations (Bennett et al., 2016). SMC-derived plaque cells comprise heterogeneous lineage-derived states with distinct structural and pathological functions, including matrix-producing cells that support cap integrity, foam-cell-like cells that accumulate lipid, and other phenotypically modulated populations with variable functional properties (Bennett et al., 2016; Pi et al., 2021). In advanced atherosclerosis, P2RY12-driven PI3K–AKT–mTOR signalling provides a disease-specific example: this pathway suppresses autophagy in SMCs, thereby promoting SMC-derived foam-cell formation and contributing to lesion progression (Pi et al., 2021). This observation supports a context-specific pathological SMC programme in the studied model, but should not be generalized to imply that mTOR activity is uniformly detrimental across all SMC-derived states. Matrix-producing SMC-derived states contribute to cap integrity (Harman and Jørgensen, 2019; Pan et al., 2020), whereas mTOR has established roles in anabolic regulation more generally (Saxton and Sabatini, 2017). On this basis, we hypothesize that mTOR activity may support matrix synthesis in selected cap-associated SMC states; a requirement for mTOR in fibrous-cap maintenance has not been directly established by these cited studies and requires state-resolved testing.

Lesion progression therefore changes the relative prevalence of cellular states rather than replacing one uniform programme with another (Libby, 2021; Gong et al., 2025). Plaque stage is consequently an imperfect surrogate for mTOR context; biological interpretation requires resolving the responding cell state within its spatial and metabolic niche, and distinguishing whether mTOR activation promotes lipid accumulation, inflammation, matrix production, or cell survival in each specific setting.

### 2.2 Complex-specific outputs and evidence asymmetry

Cell state is translated into biological output through two signalling assemblies with overlapping but non-equivalent functions. mTORC1 and mTORC2 differ in composition, upstream regulation and substrate selection (Liu and Sabatini, 2020; Szwed et al., 2021). A report of increased or decreased “mTOR signalling” is therefore mechanistically incomplete unless it identifies, as far as the data permit, the complex and downstream programme involved.

mTORC1 is the more direct integrator of amino-acid availability, growth-factor signalling and cellular energy status. Its outputs coordinate protein and lipid synthesis, metabolic adaptation and repression of autophagy (Goul et al., 2023). Canonical mTORC1 outputs include S6 kinase (S6K)- and eukaryotic translation initiation factor 4E-binding protein 1 (4E-BP1)-dependent control of translation, together with broader regulation of biosynthesis, nutrient adaptation and autophagic activity (Choo et al., 2008; Böhm et al., 2021; Goul et al., 2023). In plaque macrophages, these functions link nutrient availability to inflammatory and metabolic programmes, whereas in phenotypically modulated SMCs, these mTORC1 outputs can intersect with growth-factor signalling and autophagic control. In canonical insulin/IGF signalling models, activation of the mTORC1–S6K axis promotes inhibitory serine phosphorylation and downregulation of IRS1, thereby attenuating IRS-dependent PI3K signalling (Harrington et al., 2004; Shah and Hunter, 2006). Whether this feedback operates similarly in plaque SMC states remains to be established. Separately, mTORC1-mediated suppression of autophagy may influence lipid handling and cellular stress responses in macrophages and SMC-derived foam cells (Zhang et al., 2020; Pi et al., 2021). In a macrophage-specific Raptor-disruption model, chemokine expression and experimental atherosclerosis were reduced, indicating that selected macrophage mTORC1 programmes may promote disease in that experimental context (Ai et al., 2014). These canonical functions do not establish mTORC1 as intrinsically pathological: the biological consequence of mTORC1 activity remains dependent on substrate output, cellular state and stress context.

mTORC2 adds a distinct layer of regulation through Akt-dependent survival, cytoskeletal organization and stress adaptation (Fu and Hall, 2020). Its role is likewise context dependent rather than uniformly protective. Notably, macrophage/myeloid mTORC2 loss has not produced a uniform lesion phenotype across experimental systems. In a model involving lethally irradiated LDLR-deficient recipients reconstituted with bone marrow from mice carrying myeloid Rictor deficiency, monocyte/macrophage rapamycin-insensitive companion of mTOR (Rictor) loss reduced atherosclerosis in association with impaired monocyte/macrophage proliferation and viability (Babaev et al., 2018), whereas an independent study using non-transplanted LysM-Cre/Rictor^flox/flox^/ApoE^−/−^ mice showed in that model that mTORC2 deficiency led to significantly larger and more complex lesions—characterized by increased aortic root plaque area, expanded necrotic cores, heightened macrophage apoptosis and defective efferocytosis—driven by forkhead box O1 (FoxO1) derepression and subsequent IL-1β-dependent inflammatory signalling (Zhang et al., 2023). The opposing lesion outcomes were therefore obtained in different host models and under different experimental designs, including differences in host genotype, irradiation and bone-marrow transplantation, and the context of Rictor deletion. These findings do not establish disease stage as the cause of the discordance. Stage dependence should instead be regarded as a mechanistic hypothesis that requires direct testing within the same host model and a stage-matched experimental design.

This interpretational challenge is not unique to the macrophage lineage. Vascular studies have implicated endothelial mTOR—including mTORC2-dependent components—in nitric-oxide bioavailability, cytoskeletal regulation and adaptive responses, but these observations derive largely from non-atherosclerotic vascular injury models and have not been validated in the context of spontaneous plaque progression, endothelial barrier preservation or fibrous-cap maintenance (Fu and Hall, 2020; Wang et al., 2022; Millar et al., 2024). Thus, neither macrophage genetic evidence nor supportive vascular studies justify a universal model in which mTORC2 is uniformly protective or functions as a general vascular-repair pathway.

This imbalance in evidence strength must be explicitly acknowledged, rather than obscured by treating endothelial, macrophage and smooth muscle mTOR signalling as interchangeable pathways. To facilitate such acknowledgement, we propose a three-tier framework for appraising mTOR-related evidence in atherosclerosis. To make this hierarchy explicit, Figure 1 uses three colour-coded evidence badges. Tier one (orange) denotes direct genetic perturbation of mTOR or an mTOR-complex component with atherosclerotic lesion outcomes, as exemplified by macrophage studies targeting Raptor or Rictor (Ai et al., 2014; Babaev et al., 2018; Zhang et al., 2023). Tier 2 (purple) denotes disease-relevant mechanistic evidence linking mTOR signalling to atherosclerosis-related cellular functions without establishing the lesion effect of cell-specific genetic disruption of mTOR or an mTOR complex. The study by Pi et al. belongs to Tier 2 because it combines vascular smooth muscle cell experiments with an *in-vivo* P2RY12 intervention, rather than SMC-specific genetic deletion of mTOR or an mTOR-complex component (Pi et al., 2021). Tier 3 (blue) denotes supportive evidence from reductionist or non-atherosclerotic experimental systems, together with mechanistic inferences whose plaque-specific relevance remains unestablished; examples include immune and angiogenic models and studies of vascular homeostatic functions outside atherosclerotic plaques (Byles et al., 2013; Oberkersch et al., 2022; Wang et al., 2022; Millar et al., 2024). Evidence badges classify the supporting studies, whereas panel colours distinguish cellular states and arrow styles indicate the support for individual relationships.

**FIGURE 1:**
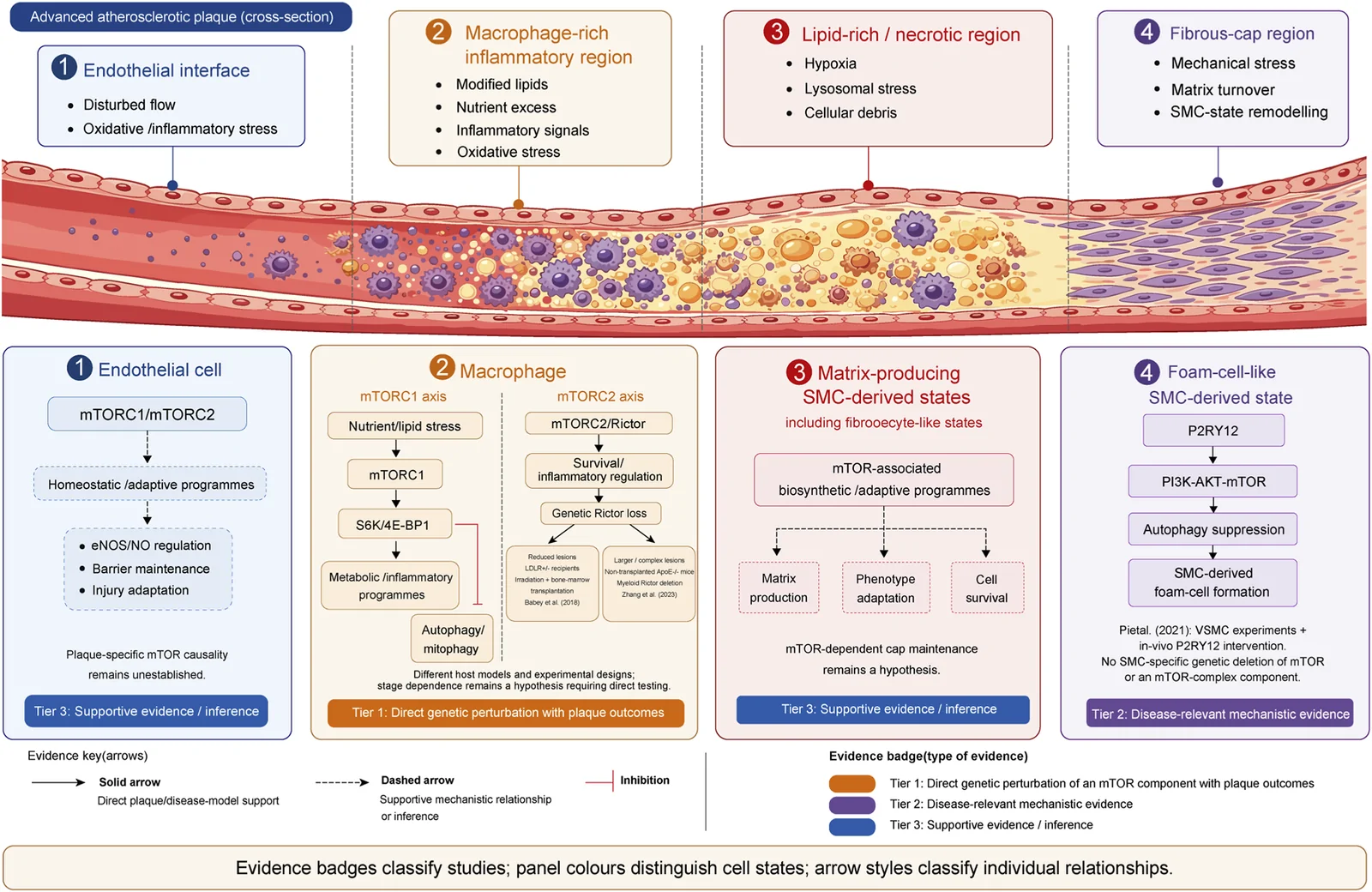
Spatial heterogeneity of mTOR signalling and cellular states across advanced atherosclerotic plaques. (AKT, protein kinase B; 4E-BP1, eukaryotic translation initiation factor 4E-binding protein 1; eNOS, endothelial nitric oxide synthase; mTOR, mechanistic target of rapamycin; mTORC1, mechanistic target of rapamycin complex 1; mTORC2, mechanistic target of rapamycin complex 2; P2RY12, purinergic receptor P2Y12; PI3K, phosphoinositide 3-kinase; Rictor, rapamycin-insensitive companion of mTOR; S6K, ribosomal protein S6 kinase; SMC, smooth muscle cell).

Conflating these evidence levels would create false symmetry between directly demonstrated plaque mechanisms and biologically plausible homeostatic functions. The key problem is therefore not a simple mTORC1-versus-mTORC2 dichotomy, but unequal and, in some settings, internally discordant evidence for complex-specific functions across plaque cell states (Figure 1).

### 2.3 Amplitude and duration dependence

mTOR inhibition is often discussed as though pathway activity were a linear dial—turn it down a little and get a little effect, turn it down a lot and get a lot of effect. Yet biological selectivity depends not simply on how much the pathway is inhibited, but jointly on how far (amplitude) and for how long (duration) signalling is displaced from its baseline. For the purposes of this Review, we distinguish homeostatic anabolic and stress-response outputs from disease-amplifying outputs associated with inflammation, lipid handling and lesion progression. This distinction represents our conceptual synthesis of general mTOR biology and selected plaque studies (Ai et al., 2014; Saxton and Sabatini, 2017; Zhang et al., 2020; Pi et al., 2021), rather than an experimentally validated binary classification. The same mTOR-regulated output may serve different functions across cell states, and neither these categories nor the cited studies establish a plaque-specific amplitude–duration boundary separating benefit from harm. Both persistent activation and profound suppression can therefore be harmful, but through different mechanisms (Saxton and Sabatini, 2017; Liu and Sabatini, 2020; Szwed et al., 2021). A dose that modifies one downstream output cannot be assumed to inhibit every mTOR-regulated process to the same degree.

This non-equivalence is evident even within mTORC1, where different substrates show markedly different sensitivities to the same inhibitory input. Rapamycin-sensitive substrates do not respond uniformly: S6K activity can remain strongly suppressed even at low drug exposure, whereas 4E-BP1 phosphorylation may be less completely or less durably inhibited, with the magnitude of this divergence varying substantially among cellular contexts (Choo et al., 2008; Böhm et al., 2021). The consequence is that a drug concentration that virtually abolishes S6K phosphorylation may leave 4E-BP1-dependent translation control largely intact. Accordingly, changes in a single downstream marker—for instance, measuring only p-S6 as a readout of mTORC1 activity—cannot be assumed to represent equivalent displacement of all mTORC1-regulated programmes. Substrate sensitivity therefore adds a quantitative dimension to context dependence: the same perturbation produces different patterns of output inhibition depending on which substrate is being measured, and this pattern varies across cell types.

Earlier work showed that leucine-mediated macrophage mTORC1 activation suppresses mitophagy, promotes the accumulation of dysfunctional mitochondria and increases macrophage apoptosis in experimental atherosclerosis (Zhang et al., 2020). Building on this mechanistic framework, the 2024 study identified a leucine-dependent threshold effect on mTORC1 activation and associated downstream responses in human monocyte-derived macrophages, including inhibition of autophagy and mitophagy. These findings extend the earlier mechanistic work by characterizing the concentration-dependent relationship between leucine exposure, macrophage mTORC1 activation and downstream cellular responses (Zhang et al., 2024). Importantly, however, the threshold identified in this study is specific to leucine stimulation, macrophage context and the experimental conditions used; it cannot be directly extrapolated to define a rapalog dose–response curve or a therapeutic window. More broadly, both insufficient and excessive mTOR activity may perturb different cellular programmes, but the boundaries between these two types of harmful responses cannot be inferred from nutrient-activation experiments alone—they require direct pharmacological intervention studies that measure drug exposure, target engagement and downstream responses in a cell-resolved manner.

Time adds yet another layer of complexity. Even when the administered concentration remains unchanged, the biological effect of mTOR inhibition can shift over time. Acute rapamycin exposure is weighted toward inhibition of rapamycin-sensitive mTORC1 substrates such as S6K, whereas prolonged treatment can gradually impair mTORC2 assembly and AKT signalling in susceptible cells (Sarbassov et al., 2006). This duration-dependent effect is itself context sensitive: rapamycin-mediated disruption of mTORC2 varies among cell types and is influenced by cellular determinants such as FKBP12 expression levels (Schreiber et al., 2015). In some cells, mTORC2 may remain intact for days; in others, it may be compromised within hours. Thus, while this observation supports the existence of a temporal dimension to rapalog pharmacology, it does not define a universal exposure duration at which mTORC2 function is lost across all vascular cells. The safe and effective duration of mTOR inhibition therefore cannot be generalized; it must be determined empirically for each cell type and context.

Amplitude and duration should consequently be considered as interacting rather than independent determinants of pathway displacement. Different combinations of perturbation intensity and duration generate different, non-equivalent patterns of substrate and complex engagement (Sarbassov et al., 2006; Choo et al., 2008; Schreiber et al., 2015; Kurdi et al., 2018). For example:

High amplitude + short duration may preferentially suppress S6K phosphorylation while sparing 4E-BP1 and mTORC2, allowing rapid recovery after drug washout.

Low amplitude + long duration may produce gradual, cumulative inhibition of S6K, with the extended exposure time increasing the risk of mTORC2 disruption despite the lower intensity.

High amplitude + long duration represents the most profound perturbation, likely suppressing both mTORC1 substrates and impairing mTORC2 assembly, with slow or incomplete recovery.

The key takeaway is that nominal administered dose alone is insufficient to describe the biological intensity of mTOR perturbation; two different dosing regimens delivering the same total drug amount may produce entirely different effects depending on how that amount is distributed over time. Similarly, acute molecular readouts—such as p-S6 or p-AKT measured a few hours after a single dose—cannot automatically be extrapolated to chronic pathway behaviour, because prolonged exposure can engage mechanisms (such as mTORC2 disruption or feedback loop activation) that are not captured by short-term measurements.

Taken together, plaque niche and cell state determine which biological programmes are present; complex identity and substrate selection determine which outputs can be engaged; and amplitude and duration determine the extent to which those outputs are displaced and whether mTORC2 becomes involved. Context dependence therefore defines why a uniform mTOR perturbation may be biologically ambiguous: the same drug, at the same nominal dose, can produce different effects in different cells and at different times. Whether this ambiguity translates into differential therapeutic benefit—or harm—must be judged from intervention studies that measure exposure, engagement and response in a cell-resolved and time-resolved manner, rather than inferred from signalling biology alone.

## 3 What existing mTOR-directed studies actually demonstrate

### 3.1 Pharmacological and lesion-directed rapalog evidence

Rapamycin and related mTOR inhibitors have generated a substantial but methodologically heterogeneous preclinical literature in atherosclerosis. Systemic rapamycin has reduced atherosclerotic progression in apolipoprotein E (ApoE)-deficient mice, including reductions in plaque progression and monocyte/macrophage-associated responses (Pakala et al., 2005), while low-dose oral sirolimus reduced atherogenesis and vascular inflammation and modified plaque composition in LDLR-deficient mice (Zhao et al., 2009). Broader experimental evidence is consistent with lesion-modifying effects of mTOR inhibition (Martinet et al., 2014; Kurdi et al., 2016; Kurdi et al., 2018). These findings establish that pharmacological mTOR perturbation can modify experimental atherosclerosis, but they do not identify the cell type, signalling complex or downstream substrate responsible for the net lesion phenotype. Systemic exposure simultaneously engages immune, metabolic and vascular compartments, and prolonged rapamycin can also produce metabolic liabilities associated with mTORC2 disruption (Lamming et al., 2012). Preclinical efficacy should therefore not be equated with a cell-selective causal mechanism.

Evidence from studies in established lesions strengthens, but also qualifies, the case for plaque-modifying effects of rapalogs. In cholesterol-fed New Zealand rabbits subjected to balloon-induced aortic injury, oral rapamycin initiated after lesion induction reduced plaque burden and inflammatory-marker expression, increased fibrous-cap thickness and lowered a histological vulnerability index (Chen et al., 2009). The reported reduction in plaque rupture should be interpreted cautiously because rupture was pharmacologically provoked with Chinese Russell’s viper venom and histamine rather than observed spontaneously. In a complementary rabbit study, everolimus-eluting stents implanted into atherosclerotic rabbit arteries produced marked plaque-macrophage depletion with relative preservation of smooth muscle cells, demonstrating that local mTOR inhibition can remodel the cellular composition of pre-existing plaques (Verheye et al., 2007), although stent implantation itself introduces vascular injury. More direct evidence for treatment of advanced disease comes from ApoE^−/−^Fbn1C1039G^+/−^ mice, in which everolimus was initiated only after 12 weeks of Western diet, when advanced lesions were already present, and continued for a further 12 weeks. Everolimus reduced circulating Ly6C^high^ monocytes and plaque macrophage content, abolished intraplaque neovascularization and was associated with improved cardiac function and survival (Kurdi et al., 2019). Together, these studies provide evidence that rapalogs can modify already established lesions rather than merely prevent lesion initiation, while their model-specific features limit direct extrapolation to spontaneous human plaque rupture.

Local vascular delivery provides a second, more spatially restricted line of evidence. Sirolimus and related drug-eluting stents (DESs) demonstrate that sustained mTOR-directed exposure can be concentrated within the arterial wall and can suppress injury-induced neointimal proliferation. However, pathological studies also document delayed healing and incomplete re-endothelialization after drug-eluting stent implantation (Finn et al., 2007; Nakazawa et al., 2008). These observations establish the feasibility—and potential biological cost—of local vascular mTOR inhibition, but percutaneous coronary intervention (PCI)-associated restenosis follows a mechanical-injury programme that is not equivalent to the evolution of a spontaneous lipid-rich plaque. Drug-eluting stents should therefore be treated as a pharmacological precedent for local exposure rather than direct validation of plaque-stabilizing mTOR therapy. More directly relevant evidence now comes from lesion-directed rapamycin delivery systems. Platelet-membrane-coated rapamycin nanoparticles preferentially accumulated in atherosclerotic lesions and reduced plaque progression while improving indices of plaque stability in ApoE-deficient mice (Song et al., 2019). Lectin-like oxidized low-density lipoprotein receptor-1 (LOX-1)-targeted rapamycin spherical nucleic acids similarly enhanced plaque-directed delivery and reduced atheroma progression with improved plaque-stability measures (Guo et al., 2022). More recently, multifunctional peptide nanoparticles designed to target plaque-associated subendothelial components and oxidized LDL delivered rapamycin to lesions and reduced foam-cell formation and local inflammatory readouts in experimental atherosclerosis (Wang et al., 2024). Additional biomimetic leukosome-based rapamycin delivery reduced aortic macrophage proliferation and pro-inflammatory signalling and altered plaque morphology after a short treatment schedule in ApoE-deficient mice (Boada et al., 2020). A 2026 study further evaluated a PP1-peptide-targeted, ultrasound-responsive rapamycin nanosystem in ApoE^−/−^ mice after 12 weeks of high-fat feeding had already established atherosclerotic plaques. Subsequent 21-day treatment enhanced plaque localization and, when combined with low-intensity pulsed ultrasound, reduced plaque and lipid area together with macrophage- and inflammation-associated readouts (Wang et al., 2026). This study extends plaque-directed rapamycin delivery into an established-lesion treatment setting, although the multicomponent formulation and ultrasound intervention complicate attribution of the lesion response to rapamycin alone.

Taken together, the intervention literature supports several distinct evidential conclusions that should not be treated as interchangeable. Systemic studies demonstrate attenuation of atherogenesis or lesion progression; rabbit and advanced-mouse studies show that rapalogs can also modify the composition of pre-existing lesions; and lesion-directed formulations demonstrate that plaque-biased drug accumulation is experimentally achievable. However, histological indices associated with stability, macrophage depletion and resistance to experimentally provoked rupture are not equivalent to demonstration of reduced spontaneous plaque rupture. These studies are important because they show that “rapamycin plus lesion targeting” is experimentally achievable rather than merely a formulation hypothesis. Their evidential endpoint, however, is still largely delivery or plaque accumulation followed by a lesion phenotype. They do not yet establish the complete sequence from local unbound rapamycin exposure to cell-specific availability, FKBP12-dependent mTOR target engagement, substrate-resolved pharmacodynamics, recovery of complex-specific signalling and plaque-level structural response. Consequently, these platforms demonstrate the feasibility of localized delivery and lesion remodelling rather than true cell-selective rapalog pharmacokinetics–pharmacodynamics (PK–PD). Thus, existing intervention evidence supports lesion modification across prevention/progression and established-disease settings, but does not establish that favourable plaque-composition changes translate into protection from spontaneous plaque rupture. An overview of the major mTOR-directed interventions, their principal findings and key limitations is provided in Table 1.

**TABLE 1:** Evidence matrix for mTOR-directed interventions in experimental atherosclerosis.

Intervention	Model/exposure context	Principal finding	Evidence supports	Major limitation
Systemic rapalogs: prevention/progression (Pakala et al., 2005; Zhao et al., 2009)	ApoE^−/−^ and LDLR^−/−^ mice; systemic treatment during developing/progressing atherosclerosis	Reduced lesion progression, inflammation and/or atherogenesis	Systemic mTOR inhibition can attenuate experimental atherosclerosis during lesion development/progression	Whole-body exposure limits cell-specific interpretation
Systemic rapalogs: established-lesion treatment (Chen et al., 2009; Kurdi et al., 2019)	Cholesterol-fed rabbits after lesion induction and ApoE Fbn1C1039G^+/−^ mice with established advanced lesions	Reduced plaque burden and inflammation; modified the composition of pre-existing lesions	Systemic rapalogs can modify established experimental atherosclerotic lesions	Whole-body exposure limits cell-specific interpretation; rabbit plaque rupture was experimentally provoked
Rapalog drug-eluting stents (Finn et al., 2007; Verheye et al., 2007; Nakazawa et al., 2008)	Atherosclerotic rabbit arteries and vascular injury/restenosis models; local arterial rapalog exposure	Reduced neointimal growth; altered plaque cellular composition, but delayed healing/re-endothelialization	Local mTOR inhibition is feasible	Stent-related injury limits extrapolation to spontaneous plaque stabilization
Plaque-targeted rapamycin systems (Song et al., 2019; Boada et al., 2020; Guo et al., 2022; Wang et al., 2024; Wang et al., 2026)	Predominantly ApoE^−/−^ mice with progressing or established atherosclerosis; lesion-directed nanoparticle delivery	Increased plaque accumulation with improved plaque-related phenotypes	Plaque-biased delivery is achievable	Drug accumulation does not establish cell-specific target engagement or pharmacodynamic selectivity

### 3.2 What remains unresolved

The pharmacological evidence reviewed above—from systemic rapamycin to drug-eluting stents to lesion-directed nanoparticles—collectively establishes that mTOR perturbation can modify experimental atherosclerosis and that plaque-biased delivery is experimentally achievable. However, it leaves four critical gaps unresolved.

First, total drug measured in a plaque does not establish the concentration of pharmacologically available rapalog at the relevant intracellular target. A plaque may contain substantial drug mass while only a fraction is available in the appropriate cellular compartment for productive target engagement. Second, whole-plaque changes in mTOR or downstream phosphorylation cannot identify which cell population was engaged; cell-resolved profiling can improve attribution (Cochain et al., 2018; Fernandez et al., 2019; Pan et al., 2020), but must be connected to pathway-specific target engagement. Third, nonlinear biological responses—including the leucine-dependent threshold behaviour of macrophage mTORC1 (Zhang et al., 2024)—support context-dependent signalling but do not define a pharmacological rapalog therapeutic window. Fourth, preferential plaque accumulation does not by itself demonstrate repair-sparing selectivity: a delivery system may increase lesion drug accumulation without establishing whether endothelial or matrix-producing SMC programmes are preserved.

Thus, existing pharmacological studies establish that mTOR perturbation can modify experimental atherosclerosis and that plaque-biased rapamycin delivery and accumulation are experimentally achievable, but they do not yet define cell-resolved exposure–engagement–response relationships. Spatially and temporally unresolved inhibition may therefore create biological-state, spatial-exposure and duration/complex mismatches. These gaps define an exposure–engagement–response problem that motivates the testable pharmacological framework summarized in Figure 2, rather than an established precision-dosing strategy.

**FIGURE 2:**
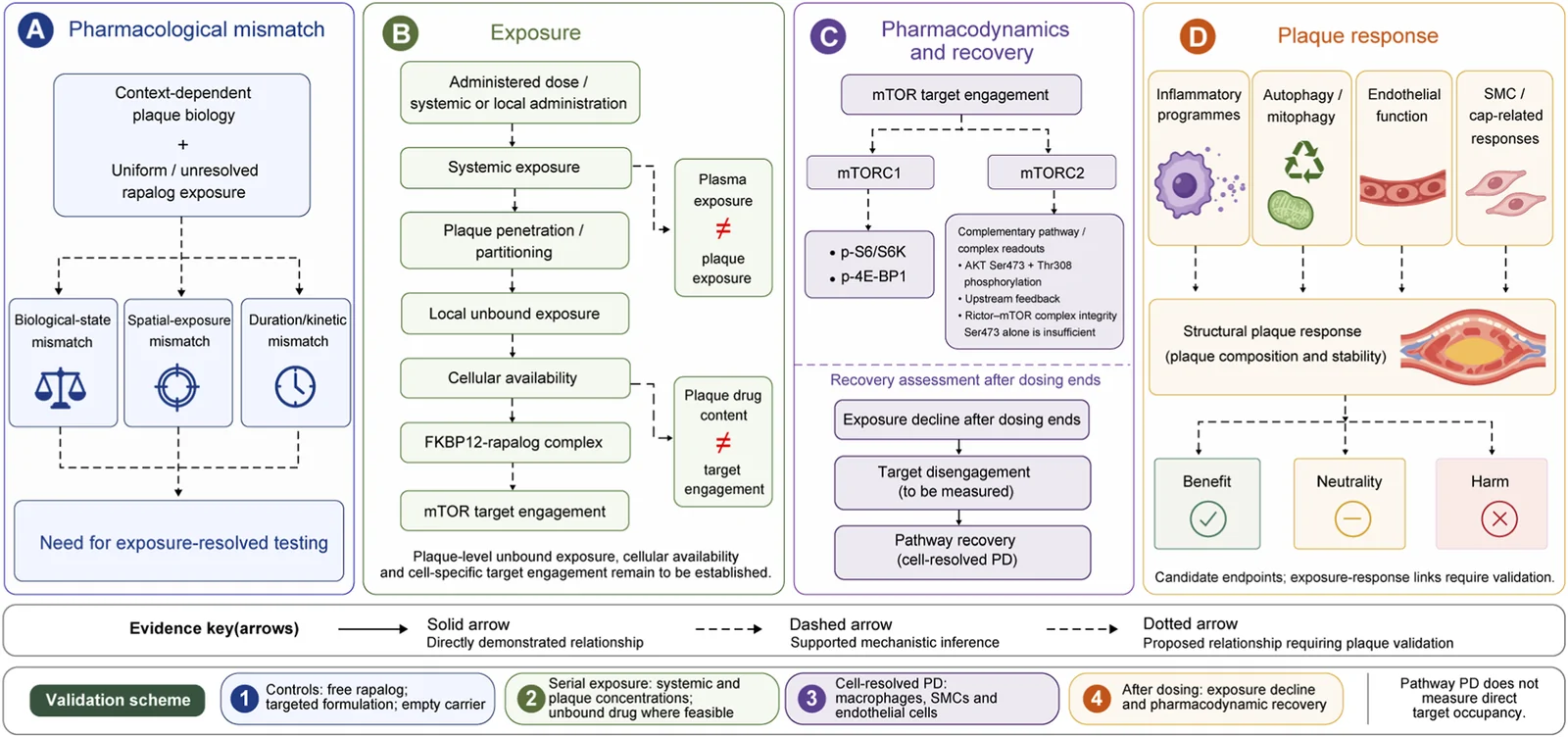
Pharmacological mismatch, exposure dynamics, and context-dependent plaque responses to mTOR modulation. (FKBP12, FK506-binding protein 12; mTOR, mechanistic target of rapamycin; mTORC1, mechanistic target of rapamycin complex 1; mTORC2, mechanistic target of rapamycin complex 2; p-AKT, phosphorylated protein kinase B; p-S6/S6K, phosphorylated ribosomal protein S6/ribosomal protein S6 kinase; p-4E-BP1, phosphorylated eukaryotic translation initiation factor 4E-binding protein 1; SMC, smooth muscle cell).

## 4 Exposure-guided mTOR modulation as a testable pharmacological framework

The framework proposed here is hypothesis-generating rather than a validated plaque PK–PD model. It identifies relationships that require experimental measurement and does not assume that their thresholds or cell-specific consequences have already been established in atherosclerotic plaques. A compact validation scheme should compare free rapalog, a targeted formulation and an empty-carrier control; measure systemic and plaque exposure at serial time points, including unbound concentrations where technically feasible; assess pharmacodynamic responses separately in macrophages, SMCs and endothelial cells; and follow exposure decline and pharmacodynamic recovery after dosing ends.

### 4.1 From administered dose to active plaque exposure

For rapalog pharmacology, administered dose represents only the beginning of the exposure pathway. Systemic pharmacokinetics (PK) determine circulating drug availability (Emoto et al., 2013), but the pharmacologically relevant sequence extends through plaque penetration and partitioning (Marlevi and Edelman, 2021), local unbound exposure, cellular uptake and cytosolic availability, FKBP12–rapalog complex formation, mTOR target engagement, substrate-specific pharmacodynamic effects and subsequent pathway recovery (Banaszynski et al., 2005; Copeland et al., 2006; Hausch et al., 2013; Lee et al., 2019; Knockenhauer and Copeland, 2024; Liu et al., 2024). Each step can alter the relationship between administered dose and biological response. Consequently, equivalent plasma exposure should not be assumed to imply equivalent plaque exposure, and equivalent plaque drug content should not be assumed to imply equivalent intracellular target engagement. Lesion architecture further complicates this relationship because extracellular matrix, lipid content, cellular density and local transport conditions can influence arterial drug distribution (Marlevi and Edelman, 2021).

At the level of target engagement, local unbound drug concentration and its time course are more informative than total tissue accumulation alone because target engagement may be influenced by drug availability, association–dissociation kinetics and intracellular target access (Copeland et al., 2006; Lee et al., 2019; Knockenhauer and Copeland, 2024). Rapamycin adds an additional molecular step: it first binds FKBP12, and the FKBP12–rapamycin complex subsequently engages the FKBP12–rapamycin-binding domain (FRB) of mTOR (Banaszynski et al., 2005; Hausch et al., 2013). Total plaque drug, total intracellular drug and mTOR target occupancy are therefore non-equivalent quantities. A plaque may contain substantial drug mass while only a fraction is available in the appropriate cellular compartment for productive target engagement.

Low aqueous solubility of rapalog interacts with heterogeneous plaque microdomains to alter drug partitioning and diffusion; generalized lipid-reservoir kinetic models lack lesion-specific pharmacokinetic (PK) validation (Marlevi and Edelman, 2021; van der Wagt et al., 2024). In particular, a generic “lipid-reservoir” model cannot substitute for direct lesion pharmacokinetic measurement. The relevant experimental objective is to determine how administered exposure is transformed into local unbound and intracellular exposure within defined plaque compartments.

An exposure-guided framework therefore separates sequential quantities that are often treated as interchangeable: administered dose, systemic exposure, plaque drug content, local pharmacologically available drug, cellular availability, target engagement and pharmacodynamic response. This separation establishes the pharmacological basis for testing selective modulation without presuming that plaque accumulation itself constitutes therapeutic selectivity.

### 4.2 A putative therapeutic window defined by engagement and recovery

A therapeutic window for plaque-directed rapalog modulation should presently be regarded as putative rather than experimentally established. Conceptually, its lower boundary would correspond to exposure insufficient to modify the intended maladaptive target output, whereas its upper boundary would correspond to exposure sufficiently deep or prolonged to perturb unintended homeostatic programmes. These boundaries cannot yet be assigned to specific macrophage, endothelial or SMC exposure thresholds because such cell-resolved relationships have not been measured directly in atherosclerotic plaques. The window is therefore best treated as a testable exposure–engagement–response hypothesis rather than a defined dosing range.

Target engagement must also be distinguished from downstream pathway response. Because target engagement depends on local exposure and disengagement kinetics, the relevant window must be evaluated using exposure and pharmacodynamic measurements rather than administered dose alone (Copeland et al., 2006; Lee et al., 2019; Knockenhauer and Copeland, 2024; Liu et al., 2024). Rapamycin-sensitive mTORC1 substrates are not equally inhibited: ribosomal protein S6 (S6)/S6K phosphorylation is generally more rapalog sensitive, whereas 4E-BP1 phosphorylation can show incomplete or kinetically distinct suppression (Choo et al., 2008; Böhm et al., 2021). Accordingly, p-S6 or p-S6K and p-4E-BP1 can serve as complementary PD indicators of mTORC1 pathway displacement. AKT Ser473 phosphorylation alone cannot establish rapalog-induced mTORC2 disruption, although prolonged rapamycin exposure may impair mTORC2 assembly and reduce AKT Ser473 phosphorylation (Sarbassov et al., 2006; Schreiber et al., 2015). Relief of mTORC1/S6K-mediated negative feedback can increase upstream PI3K signalling and thereby increase AKT phosphorylation (O'Reilly and McSheehy, 2010). Assessment of mTORC2 disruption should therefore combine AKT Ser473 with AKT Thr308 phosphorylation, upstream feedback measurements such as IRS1 and PI3K–PDK1 signalling, and biochemical evaluation of Rictor–mTOR complex integrity, for example by co-immunoprecipitation. None of these markers alone measures intracellular rapalog target occupancy or predicts plaque benefit, endothelial repair or fibrous-cap preservation. These phosphorylation and complex-integrity measurements are pharmacodynamic pathway readouts rather than direct measurements of drug–target occupancy. Direct target occupancy would require a validated binding or competitive target-engagement assay capable of quantifying productive FKBP12–rapamycin–mTOR engagement. Their value lies in defining downstream pathway displacement and recovery, which must then be related separately to cell-state and structural outcomes.

Intermittent or pulse exposure is consequently a testable strategy rather than an established solution. Any advantage of such an approach would require at least four conditions: the intended pathological output must be measurably suppressed; local pharmacologically active exposure must subsequently decline; the relevant homeostatic signalling programmes must recover after exposure falls; and repeated administration must not accumulate into quasi-continuous target occupancy. These conditions depend on both local pharmacokinetics and pharmacodynamic recovery rather than on the nominal dosing interval alone (Copeland et al., 2006; Lee et al., 2019; Knockenhauer and Copeland, 2024; Liu et al., 2024). None of these conditions has yet been established in atherosclerotic plaques.

The central experimental question is therefore whether exposure decline, target disengagement and pathway recovery can be temporally separated from the desired period of pathway modulation. Demonstrating such separation would provide evidence for a pharmacological window; until then, “therapeutic window” should denote a hypothesis to be measured rather than a property assumed from dose–response or duration studies.

### 4.3 Spatial bias and pharmacology–pharmaceutics co-design

Spatial bias adds a second dimension to exposure control. Three broad strategies are pharmacologically distinct: lesion-biased systemic delivery seeks to increase plaque relative to systemic exposure; cell-biased uptake seeks to preferentially increase intracellular exposure in selected lesional populations; and local vascular delivery seeks to restrict drug deposition to a defined arterial region (Finn et al., 2007; Nakazawa et al., 2008; Korin et al., 2012; Marlevi and Edelman, 2021; Fu et al., 2026). Cell-biased uptake does not itself establish cytosolic rapalog availability because intracellular trafficking and payload release may limit access to FKBP12 and mTOR. None of these strategies guarantees cell-selective target engagement. Increased plaque accumulation may coexist with broad distribution across plaque compartments, whereas enhanced uptake by one cell population may still leave substantial extracellular or systemic exposure. Spatial bias should therefore be assessed by exposure distribution rather than total lesion drug content alone. Haemodynamics, endothelial permeability, lesion composition and cellular uptake can each influence local drug distribution (Korin et al., 2012; Marlevi and Edelman, 2021; Fu et al., 2026). These determinants should be treated as sources of exposure heterogeneity rather than as evidence that any specific targeting mechanism is intrinsically beneficial. For example, improved localization to a diseased endothelial interface does not establish that intracellular rapalog release in endothelial cells is desirable, and greater plaque retention does not establish preferential engagement of the intended cell state. Spatial targeting and biological selectivity are therefore related but non-equivalent properties.

Existing lesion-directed rapamycin systems demonstrate that plaque-biased delivery and accumulation are experimentally achievable, but have not yet established compartment-resolved free-drug pharmacokinetics or cell-specific mTOR target engagement (Song et al., 2019; Guo et al., 2022; Wang et al., 2024). This distinction is essential: delivery or accumulation followed by an improved plaque phenotype establishes feasibility, but does not identify the intracellular exposure profile responsible for that phenotype. Other plaque-targeted nanomedicines provide proof of principle that lesion- or cell-biased delivery can be achieved (Flores et al., 2020; Gao et al., 2023; Bamezai et al., 2024; Fang et al., 2025; Li et al., 2025), although their pharmacodynamic kinetics cannot be directly extrapolated to FKBP12-dependent rapalog target engagement.

For rapalogs, formulation can therefore shape the pharmacodynamic profile by altering where pharmacologically active drug appears, how much becomes intracellularly available and how long target engagement persists. Pharmacology and pharmaceutics should consequently be co-designed around a limited set of measurable variables rather than a large set of assumed kinetic parameters. Five variables are particularly informative: the lesion-to-systemic exposure ratio, cellular uptake distribution and cytosolic payload availability, local unbound exposure, duration of target engagement and pathway recovery time. Together, these measurements would provide a minimum dataset to test whether spatial bias translates into meaningful pharmacological selectivity. Accordingly, formulation effects should be evaluated experimentally rather than represented by an unvalidated kinetic model. PK–PD and, where appropriate, physiologically based modelling can integrate lesion/systemic exposure, cellular distribution, local unbound concentration, target-engagement duration and recovery kinetics (Zou et al., 2020; Ozbek et al., 2024). The objective is not to maximize any single parameter, but to determine whether a formulation changes the relationship between administered dose and cell-resolved pharmacodynamic response in a reproducible manner.

Taken together, exposure-guided mTOR modulation can be framed as a testable sequence linking pharmacological mismatch with administered dose, plaque exposure, cellular availability, FKBP12-dependent mTOR target engagement, substrate-specific pharmacodynamics, pathway recovery and plaque response (Figure 2). The framework does not assume that spatial targeting, intermittent exposure or any particular formulation is beneficial; rather, it identifies the measurements required to determine whether pharmacological selectivity can actually be achieved.

## 5 Translational testing and boundaries

### 5.1 Matching translational context to candidate exposure objective

Translation of exposure-guided mTOR modulation should begin by defining the translational contexts in which a particular exposure objective could be biologically justified rather than proposing universal lesion-targeted dosing regimens. High-risk plaques are heterogeneous in morphology, cellular composition and biological activity, and no single mTOR-related programme can be assumed to dominate across all lesions (Cochain et al., 2018; Pan et al., 2020; Gaba et al., 2023; Vergallo et al., 2025). Two core lesion phenotypes guide experimental stratification: inflammation-dominant lipid-rich plaques and repair-fragile thin-cap plaques. These lesion-level contexts, together with inter-lesion heterogeneity as a patient- or vascular-tree-level constraint, should be regarded as translational testing conditions rather than established therapeutic indications.

In inflammation-dominant lipid-rich plaques, macrophage inflammatory and metabolic activity, defective resolution and necrotic-core progression represent the principal biological concerns (Moore et al., 2018; Fernandez and Giannarelli, 2022; Adkar and Leeper, 2024). A candidate exposure objective would therefore prioritize suppression of maladaptive inflammatory outputs while minimizing prolonged engagement of non-target programmes. Whether such an exposure profile produces the intended cell-resolved pharmacodynamic response remains to be established.

In repair-fragile thin-cap plaques, the relevant concerns shift toward cap integrity, endothelial continuity and the maintenance of SMC-derived matrix-producing states (Harman and Jørgensen, 2019; Yurdagul, 2022; Vergallo et al., 2025). This context requires particular attention to whether sustained pathway suppression perturbs repair-associated programmes and should therefore primarily serve as a safety-testing context. Current evidence does not justify specifying a “low-dose”, “cap-sparing” or otherwise optimized rapalog regimen; the appropriate question is whether a candidate exposure profile preserves repair-associated structural and cellular readouts while retaining the intended pharmacodynamic effect.

A third translational barrier stems from inter-lesion heterogeneity. Plaques with different inflammatory, lipid and structural features may coexist within the same vascular disease burden (Gaba et al., 2023; Vergallo et al., 2025), making a single lesion-agnostic exposure rule difficult to justify. Experimental studies should therefore determine whether an exposure profile optimized for one lesion context produces unintended engagement in lesions with different biological requirements. Restenotic lesions post-intervention represent a unique pharmacological niche and cannot be equated to spontaneous vulnerable plaque stabilization (Joner et al., 2006; Finn et al., 2007; Nakazawa et al., 2008). These lesion-level contexts and the patient-level constraint are summarized in Table 2 as candidate exposure objectives and experimental readouts rather than clinical treatment recommendations.

**TABLE 2:** Candidate exposure objectives and translational readouts across plaque and inter-lesion contexts.

Translational context	Dominant biological concern	Candidate exposure objective	Candidate readouts	Current evidence gap
Inflammation-dominant lipid-rich plaque (Zhang et al., 2020; Zhang et al., 2024)	Macrophage activity; defective resolution; necrotic-core progression	Test whether exposure can be biased toward inflammatory plaque compartments while minimizing prolonged non-target engagement	Cell-resolved pharmacodynamic (PD) readouts; autophagy/mitophagy; inflammatory activity; necrotic-core burden	Plaque targeting ≠ selective target engagement (Song et al., 2019; Guo et al., 2022)
Thin-cap repair-fragile plaque (hypothesis-based safety scenario) (Fu and Hall, 2020; Oberkersch et al., 2022; Wang et al., 2022; Millar et al., 2024)	Cap integrity; endothelial continuity; SMC/matrix preservation	Test whether sustained exposure compromises repair-associated signalling	Direct experimental plaque readouts: collagen content, fibrous-cap thickness and SMC content/α-SMA staining (Chen et al., 2009; Zhao et al., 2009). Proposed safety readouts: cell-resolved PD, endothelial continuity and lineage-resolved SMC state; these have not been validated as indicators of a repair-sparing exposure window in spontaneous thin-cap plaques	Repair-sparing exposure remains a safety hypothesis. The cited endothelial and stent studies provide indirect safety support from settings other than spontaneous thin-cap plaques and do not establish a plaque-specific exposure threshold (Finn et al., 2007; Nakazawa et al., 2008)
Mixed/inter-lesion heterogeneity (Mahdinia et al., 2023; Gong et al., 2025)	Heterogeneous inflammatory, lipid, and structural features	Test whether lesion-biased exposure can be achieved without excessive off-target vascular or systemic engagement	Plaque phenotype; lesion/systemic exposure; inter-lesion response; systemic PK	No validated lesion-agnostic exposure rule (Marlevi and Edelman, 2021)

### 5.2 Translational pharmacodynamic and imaging endpoints

Demonstrating plaque accumulation is insufficient to establish pharmacological selectivity. Translational evaluation should instead distinguish and relate exposure to target engagement, downstream pharmacodynamics and lesion-level response (O'Reilly and McSheehy, 2010; Simon et al., 2013). These readouts operate at different biological scales and should not be treated as interchangeable surrogate measures.

At the molecular and tissue level, p-S6/S6K and p-4E-BP1 can provide complementary pharmacodynamic readouts of mTORC1 pathway displacement, whereas assessment of mTORC2 should combine AKT Ser473 with AKT Thr308 phosphorylation, upstream feedback signalling and Rictor–mTOR complex integrity (Sarbassov et al., 2006; Choo et al., 2008; O'Reilly and McSheehy, 2010; Böhm et al., 2021). AKT Ser473 phosphorylation should not be interpreted in isolation because relief of mTORC1/S6K-mediated negative feedback may increase upstream PI3K–AKT signalling even when prolonged rapalog exposure disrupts mTORC2 assembly. Autophagic and mitophagic readouts may further connect pathway perturbation to disease-relevant cellular responses (Zhang et al., 2020; Zhang et al., 2024). Where technically feasible, these measurements should be resolved by cell type rather than derived solely from whole-plaque lysates. Endothelial readouts, including eNOS expression or phosphorylation and measures of barrier integrity, should likewise be regarded as hypothesis-generating endpoints rather than validated biomarkers of plaque benefit, because direct endothelial-specific evidence linking mTOR manipulation to spontaneous atherosclerotic plaque outcomes remains sparse and is derived mainly from non-atherosclerotic vascular injury models, as discussed in Sections 2.1, 2.2. Importantly, p-S6, p-4E-BP1 and p-AKT are PD indicators, not validated biomarkers of plaque benefit, inflammatory resolution or vascular repair. Likewise, Rictor–mTOR complex integrity reflects complex assembly rather than direct rapalog target occupancy. Direct target-occupancy measurements should therefore be distinguished explicitly from downstream phosphorylation and complex-assembly readouts. Their principal near-term role is therefore preclinical and tissue based: to establish whether a measured exposure produces a defined pathway response and whether that response reverses as exposure declines.

Plaque-level endpoints provide complementary structural and biological information. Optical coherence tomography (OCT) can assess fibrous-cap and luminal morphology; intravascular ultrasound (IVUS) can quantify plaque burden and volume; near-infrared spectroscopy (NIRS) can characterize lipid-rich plaque components; computed tomography angiography (CTA) can assess plaque burden and compositional features; and positron emission tomography (PET) can provide information on selected aspects of plaque biological activity (Kuku et al., 2020; Legutko et al., 2022; Gaba et al., 2023; Nakahara et al., 2023; Vergallo et al., 2025). No single modality captures all dimensions of high-risk plaque biology, and multimodal assessment should therefore be viewed as complementary rather than hierarchical.

Linking measured exposure and appropriately resolved molecular pharmacodynamics to serial plaque imaging could therefore test whether defined pathway displacement is associated with subsequent structural or biological plaque change. Such studies would establish exposure–pharmacodynamic–plaque response relationships rather than provide a real-time dosing algorithm.

### 5.3 Safety boundaries and evidence required for translation

Any translational programme for plaque-directed rapalog modulation must resolve four boundaries before efficacy claims can be extended beyond experimental models. First, systemic exposure remains a fundamental safety constraint. Rapamycin and its analogues can affect immune and metabolic homeostasis, with sustained exposure associated with immunosuppressive effects, glucose dysregulation and dyslipidaemia (Lamming, 2016; Saxton and Sabatini, 2017; Kurdi et al., 2018). The magnitude of these risks may vary with the compound, dose and exposure schedule. A lesion-directed formulation must therefore demonstrate not only plaque delivery but also acceptable systemic exposure and safety.

Second, spatial selectivity is unlikely to be absolute. Lesion-biased or local delivery can alter drug distribution but cannot be assumed to eliminate exposure of adjacent vascular or systemic compartments (Lobatto et al., 2011; Marlevi and Edelman, 2021; Cheng et al., 2023). Measurements of biodistribution, local unbound exposure and non-target tissue exposure are therefore required to distinguish preferential localization from genuine pharmacological selectivity.

Third, mouse plaque pharmacology cannot establish human lesion penetration, washout, cellular partitioning or pathway-recovery kinetics. Large-animal studies provide an intermediate setting in which vessel dimensions, lesion architecture and delivery performance can be examined at a more translational scale (Marlevi and Edelman, 2021; Bamezai et al., 2024), provided that the selected model reproduces the lesion features relevant to the proposed intervention. However, rapalog-specific compartment-resolved PK–PD will still require direct validation. Human tissue and translational studies will ultimately be needed to determine whether the exposure–engagement relationships inferred from experimental models are preserved in clinically relevant lesions.

Fourth, demonstration of target engagement and favourable plaque imaging does not establish clinical event reduction. Molecular target engagement, downstream pharmacodynamics, plaque-level structural responses and major adverse cardiovascular events represent distinct evidential levels (Gaba et al., 2023; Sarraju and Nissen, 2024; Vergallo et al., 2025). Clinical translation would therefore require a staged programme linking exposure first to pharmacodynamics, then to reproducible plaque-level responses and ultimately to clinically meaningful outcomes.

The near-term objective is therefore not clinical implementation of a precision mTOR dosing algorithm, but experimental definition of the plaque contexts, exposure profiles and pharmacodynamic boundaries in which selective modulation is biologically feasible.

## 6 Conclusion

mTOR signalling in atherosclerotic plaques is context dependent rather than uniformly pathogenic or protective. Its biological consequences vary according to cell state, plaque niche, signalling-complex identity, substrate selection and the amplitude and duration of pathway perturbation. Current evidence establishes that genetic and pharmacological manipulation of mTOR can modify experimental atherosclerosis, but the strength of evidence remains uneven across cell types and signalling complexes. In particular, direct lineage- and complex-resolved studies support complex-specific effects in macrophages, whereas selected SMC-state studies provide disease-relevant mechanistic support. Existing lesion-directed rapalog studies further demonstrate that plaque-biased delivery and accumulation are feasible in experimental models, but they do not define a cell-resolved pharmacodynamic or therapeutic window or quantitative relationships linking active lesion exposure to target engagement, substrate-specific pharmacodynamics, pathway recovery and plaque-level structural response. Nor has it been established that lesion targeting or intermittent exposure preserves vascular repair. Exposure-guided modulation should therefore be viewed as a testable pharmacological framework rather than an established precision-therapy strategy. Compartment-resolved pharmacokinetics, appropriately resolved target-engagement measurements, cell-resolved pharmacodynamics, recovery kinetics and validation in relevant large-animal models and human lesions are now required. The critical next step is not to assume that stronger or more localized mTOR inhibition is beneficial, but to define directly the exposure–engagement–response relationships that determine whether rapalog-mediated mTOR modulation produces benefit, neutrality or harm within the evolving plaque.
